# State of the Art, Trends and Future of Bluetooth Low Energy, Near Field Communication and Visible Light Communication in the Development of Smart Cities

**DOI:** 10.3390/s16111968

**Published:** 2016-11-23

**Authors:** Gonzalo Cerruela García, Irene Luque Ruiz, Miguel Ángel Gómez-Nieto

**Affiliations:** Department of Computing and Numerical Analysis, University of Córdoba, 14071 Córdoba, Spain; iluque@uco.es (I.L.R.); mangel@uco.es (M.Á.G.-N.)

**Keywords:** NFC, BLE, VLC, IoT, IoE, smart city

## Abstract

The current social impact of new technologies has produced major changes in all areas of society, creating the concept of a smart city supported by an electronic infrastructure, telecommunications and information technology. This paper presents a review of Bluetooth Low Energy (BLE), Near Field Communication (NFC) and Visible Light Communication (VLC) and their use and influence within different areas of the development of the smart city. The document also presents a review of Big Data Solutions for the management of information and the extraction of knowledge in an environment where things are connected by an “Internet of Things” (IoT) network. Lastly, we present how these technologies can be combined together to benefit the development of the smart city.

## 1. Introduction

Currently, more than 50% of the world’s population lives in urban areas, and it is estimated that by 2050, 66% of the world’s population will be urban [1]. This has converted many cities into highly complex systems to manage and maintain. Today’s cities, and those of the future, are systems that require government input to establish the necessary means to provide citizens with services in an equal and sustainable manner, as well as ensuring quality of life.

In order to guarantee these objectives, cities must be “smart”. There is no single definition of a smart city and different organizations each have their own definitions [2]. In [3] a smart city was defined as a “city seeking to address public issues via Information and Communication Technology (ICT)-based solutions on the basis of a multi-stakeholder, municipally based partnership”. Most standards bodies define the smart city as “innovation, not necessarily but mainly, based on ICT, which enhances urban quality of life in terms of people, governance, environment, mobility, economy, urban sustainability and living” [4].

However, all of them refer to a system of people who are interacting and using energy flow, materials, services and finance in order to enable sustainable economic development, adaptation and quality of life. These interactions and the flow of energy become “smart” by creating strategic usage of infrastructure, communication and information services through a process of planning and transparent urban management that respond to the social and economic needs of society.

Nowadays, new technologies have enormous social impact, creating major changes in industrial sectors and in all areas of society. Technological advances and the increase in smart device usage confirm the trend towards the “Internet of Things” (IoT) [5,6], which is focused on people, processes and objects. This concept has now been named more broadly as “Internet of Everything” (IoE), meaning the useful information available to people generates value rather than just communication between things.

Smart cities are an example of how the use of IoT will help make objects smart via a combination of devices, sensors, advanced communication networks, management platforms, cloud storage, etc. [7]. This improves the quality of life of citizens, increasing business opportunities, allowing for the emergence of new market tendencies and making services more efficient and sustainable.

The European Commission estimates that by 2020 there will be between 50 and 100 billion systems connected to the Web [8]. It is also predicted that the number of “things” connected to the internet will exceed the world population of 2008 [9]. In 2016, it is expected that the global market related to smart cities, will exceed a trillion dollars with an annual rate of increase of 14.2%. The construction of these new smart cities requires solutions for sustainability in three major systems:

- Urban Mobility: This requires solutions in alternative and clean energy, cleaner public transport, efficient logistics and city planning.
- Environment and Living Conditions: With the aim to improve the efficiency of cities, neighborhoods and houses, it is necessary to increase the use of renewable energy, reduce consumption, prepare for changes and integrate them into city resources, etc.
- Management of resources: This improves the integration between infrastructures and processes through new IT and communication technologies for use in energy and transport.

Undeniably, the success of the development of a smart city also requires the development of an appropriate infrastructure of information and communications, using a common platform that enables gathering, measurement and analysis of data and the monitoring, optimization and control of all the systems involved [10].

The development and installation of information and communication technologies are the basis of a smart city, integrating the six action groups via a system of platforms that manage sensors, services and products and communication between them. These platforms constitute what has been called “The Internet of the Future” which encompasses [11,12,13]:

- The Internet of Things (IoT), that enables integration between people and any element or object present in the cities.
- The Internet of Services (IoS), that enables the integration between services that manage the different systems under the same ontology, enabling interoperability, whilst managing multiple sources of information.
- The Internet of People (IoP), that enables cities to take advantage of people’s knowledge as a network of interconnected global knowledge, whilst sharing their environment and status within the context of the city.

It is clear that the development of smart cities is based on the development of Smart Connected Communities (SCC) [14] that are supported by an infrastructure of electronics, telecommunications and information technology. Electronics will allow each object, and indeed each person, to communicate via devices that are able to interact with other objects or people. Telecommunications will enable a city to be totally connected between objects and people. Information technology will allow this flow of information to be accessed, vast quantities of data to be analyzed and help establish better city planning.

The development of SCC has been able to take place due to wireless communications. The evolution of mobile technology, the reduction in price of mobile devices and their spread throughout the world will mean that in the next 50 years or so, these SCC will be completely connected.

Protocols for Machine to Machine (M2M) communication such as *LoRaWAN*, *Sigfox*, *Weightless*, *LTE*, and *5G* are considered to be one of the key enablers for the provisioning of advanced applications and services in smart cities.

The Low-Power Wide Area Networking (LP-WAN) platforms *LoRaWAN* [15], *Sigfox* [16], *Weightless* [17] have as a fundamental characteristic being scalable systems, usually in an operated fashion, employing low-cost edge-devices with low battery consumption. A review of the state of the art in LP-WAN solutions for industrial IoT services can be found in [18].

The LTE protocol enhancement the issues of overload control, network support for M2M devices, device cost reduction, power saving for ultra-long battery life, and coverage enhancement [19,20,21].

Emerging technology 5G communication is a particularly good fit for M2M communication and IoT applications by offering lower cost, lower energy consumption and support for a very large number of devices [22]. This protocol supports a large amount of mobile data traffic and a massive number of wireless connections. The 5G network is energy-efficient as well as offering quality of service in terms of communication delay, reliability, and security [23].

Other wireless technologies like Bluetooth Low Energy (BLE) or Near Field Communication (NFC) have developed and matured greatly. By incorporating the above elements into programmable systems, BLE Chips, NFC Tags or even System on Chips (SoC) a solution to the construction of a totally connected city is provided. In this way, any object or person can be connected to their environment and communicate with other objects and people via wireless communications and thereby serve as a source of information for the SCC.

Furthermore, in recent years, a new communication technology based on visible light has been developed. VLC (Visible Light Communication) enables an LED illumination infrastructure placed in an object or space to communicate wirelessly. The use of such technology eliminates any broadband width problems that occur with WiFi technology in indoor spaces, thus allowing a large number of users. VLC avoids security problems in sensitive zones where RFs can be accessed, as well as problems with communication in open zones and with mobile objects, etc. VLC presents itself as the best option for sustainability in cities, decreasing CO_2_ emissions and energy consumption, whilst favoring communication of SCCs at any time, in any place.

In Section 2, this paper presents a background of these three wireless communication technologies, BLE, NFC and VLC and their characteristics, present state of development and maturity. Section 3, describes the research and development carried out with NFC, BLE and VLC technologies, the different projects and prototypes implanted in different cities around the world, and how the future of smart cities will be influenced by the development of these three technologies. Finally, we summarize the main conclusions.

## 2. Background

In this section, we describe three emerging technologies that surround us in the network of communications and we identify and collect the interactions of their users.

### 2.1. Near Field Communication (NFC)

Near Field Communication (NFC) provides a new wireless technology that enables short distance communications and low level energy consumption between “objects” in the environment, which is the basis of IoT.

NFC is an evolution of Radio Frequency Identification (RFID) technology, created mainly for use by mobile devices. Its development was started in 2002 when Philips and Sony decided to create a protocol that would be compatible with existing technologies, which gave rise to Philips’ Mifare and Sony’s FeliCa systems [24].

The NFC protocol was trialled in 2003 with the ISO 18092 standard. In 2004, Philips, Sony and Nokia created the NFC-Forum, managing to get more than 150 companies such as Google, PayPal and Visa, amongst others, to participate in the consortium. The purpose was to provide a simple method of communicating between two electronic devices. This resulted in contactless transactions, access to digital content and the ability of electronic devices to connect with a simple touch. This was all made possible due to the fact that NFC complemented other wireless technologies, using key elements of existing regulations for contactless card technology, such as ISO/IEC 14443 A&B y JIS X-6319-4 [25,26].

NFC communicates through radio waves like RFID but with a shorter range of around a maximum distance of 14 cm (which in reality is less than 4 cm) and with a maximum transfer velocity of 424 Kbps of communication in the unregulated radio frequency ISM band of 13.56 MHz [27].

Depending on the types of interactions made, NFC devices have different work modes [28]:

- Active mode of communication: Both devices generate a radio frequency (RF) signal to transmit data without the need to pair, as is the case with other technologies.
- Passive communication mode: Only one of the devices generates the radio frequency field. The second device, the passive one (named NFC Tag), acts as a receptor and uses a technique called “charge modulation” to transfer information coming back from the active device or initiator.

There are three types of communication:

- Card emulation is used when the NFC device works as a contactless card and can be used to manage payment systems based on different methods such as Mifare, Visa payWave, MasterCard Pay Pass or American Express Express Pay.
- Reader/Writer is used to modify, store or read data from a passive element or NFCTag. In the case of reading, the active device, which receives the information, may be configured to carry out an action depending on the type of information read.
- Peer to Peer is specifically for active NFC devices, establishing a link between two devices, generally mobile phones (smartphones), which allows an exchange of information between the two.

#### 2.1.1. Advantages and Disadvantages

NFC’s strong points are its flexibility, ease of use, independence of devices and methods of communication and speed of communication, which is practically instantaneous, without the need to pair devices, setting it apart from other wireless communication technologies, such as Bluetooth.

Its energy consumption is minimal and due to its short range, communications are very secure. However, its low transmission speed (424 Kbps) does not allow for large files transfers, although this inconvenience is made up for by its ability to intercommunicate with other technologies (WiFi, Bluetooth) that do permit this. Because the connection between devices is peer to peer, NFC cannot create wireless networks between different devices.

Nevertheless, the vast number of NFC chips with specific characteristics that enable interconnection with micro-controllers and their versatility of size and the supported material in which these chips can be produced has given rise to NFC’s integration into all types of wearable devices made to give support to different IoE applications.

#### 2.1.2. Hardware and Software

The types of elements that are possible in an NFC system are:

- Mobile devices with NFC: Smartphones are the most well-known and used mobile devices, although they are not the only ones, as we have mentioned above. They allow us to take advantage of different options offered by NFC ecosystems and normally act as active elements within the NFC system.
- NFC Readers: These devices capable of accessing information from other NFC devices and sending it to other elements for processing. They are also in active mode during interactions.
- NFC Tags: These are RFID tags without an integrated feed source. They are in passive mode within the NFC system.

In an NFC system, the initiator of communication is always an element that functions in active mode and the receptor can be an element that functions in active or passive mode, such as an NFC tag, depending on the interaction that is taking place.

In order for the exchange of data to take place between two devices or an NFC tag, a standardized format has been created by the NFC Forum called NFC Data Exchange Format (NDEF) that allows storage and transport of different types of information, such as Multipurpose Internet Mail Extensions (MIME) messages, Record Type Definition (RTD) or smaller sizes such as Uniform Resource Locators (URLs) [25].

A classification has been established for NFC tags by the NFC-Forum that provides the specifications necessary for the correct interoperability between the different tag providers and the manufacturers of NFC devices. This guarantees a consistent and satisfactory experience for users, the objective being to describe how messages should be read and written in NFC tags. Based on this classification, there are five types with different characteristics [29]:

- Type 1: This is based on the ISO/IEC 14443A standard. It is for reading or writing with the option to block for read-only. Storage size is 96 bytes, expandable to 2 Kbytes. The transmission speed is 106 Kbps [30].
- Type 2: This has similar characteristics to Type 1 but its storage size is different (48 bytes expandable to 2 Kbytes) [31].
- Type 3: This is based on the Japanese industrial standard JIS X 6319-4 (Felica). It is pre-configured to be for read/write or read-only during the manufacturing stage. This type can reach up to 1 Mbytes [32].
- Type 4: This is compatible with the ISO/IEC 14443A&B standard and can be manufactured for read/write or read-only. The difference between these standards is the modulation and initialization of transmission speeds, which can reach up to 32 Kbytes of storage [33].
- Type 5: This is used for storing NDEF messages in Picopass 2K/32K and Picotag 2K, using the ISO14443B-3 protocol and supported by the ISO/IEC 15693 (NFC-V) standard that allows interaction with RFID tags [34].

An extensive collection of products is on the market that satisfies the NFC-Forum specifications and is manufactured with their usage in mind. Amongst the different tags, we can find Topaz, Mifare 1K/4K, Ultralight/C, NTag20X, Ntag21X, etc., that are available in large ranges of size and types (inlays, epoxy, keyfogs, keychain, wristbands, rings, etc.), of which the main difference is found in the type of tag or specification that they support, their storage capacity and their security.

Table 1 shows a summary of the characteristics of some of these Tags. The memory size, transmission speed, security and data protection as well as, in many cases, the minimum chip size, determine their use in different IoT applications. There is a sixth group of tag types (Type 6) that operates under the ISO/IEC 15693-3 and protocol and is geared towards identification cards [35].

Recently, new types of chips have emerged that are generically named NTAG IC [36]. These chips comply with the NFC-Forum specifications for Type 2. With a memory size of between 144 and 1904 bytes, they incorporate a contact interface I^2^C [37] that enables rapid communication of the chip without contact with a micro-controller and vice-versa. It can even feed external devices with low level energy consumption. These NTAG IC [38] chip characteristics make them ideal for IoE applications by communicating applications with the micro-controllers of household appliances, vehicles and all types of electronic appliances [39].

New technologies in printing and materials have allowed for a recent development of a new type of Tag, called NFC Barcode (Kovio Tags) [40]. The NFC Barcode is an integrated circuit board that works in Tag-Talks-First (TTF) mode, transmitting the stored code repeatedly, whilst being fed by the reader at a speed of 106 Kb/s and without the need to be activated by a single command from the reader. This tag has a storage capacity of 128 bits and operates under the ISO/IEC 14443-3 standard. These chips are ideal solutions for identification and authentication.

We can find different manufacturers for NFC chips on the market, such as NXP, Inside secure, Broadcom, Infineon, etc. and a large number of manufacturers and distributors (mainly in Asia) of Tags over a wide range of supports.

Since 2007, the number of mobile devices that include this technology has increased dramatically. Currently, three out of four smartphones made incorporate NFC [41] and also most tablets, although Apple has decided to block the NFC function on their devices so they can only be used for mobile payment operations with Apple Pay.

#### 2.1.3. Current Status

NFC technology is currently available to any end user. NFC Tags can be acquired easily and can be personalised for some of the multiple mobile applications in Google Play, such as TagWriter, NFC Tools, NFC TagInfo, NFC Writer, etc. and desktop applications such as GotoTags [42] or Sony [43].

From their side, the developers have different environments and libraries at their disposal for the designing of applications with NFC. Among these are: Open NFC that provides a unified API to access all cards compliant with NFC Tags containing NDEF messages as defined by the NFC Forum, Card Emulation mode supporting ISO 14443 4-A and ISO 14443 4-B cards, peer-to-peer communication supporting Type F and optionally Type A protocols, management of smartcard chipsets (secure element and UICC) and connection handover mechanism for Bluetooth and WiFi Pairing [44]; LibNFC a public library under GNU license supporting ISO/IEC 14443 A and B, FeliCa, Jewel/Topaz tags and P2P [45]; Android NFC API supporting NFC-A, B, F, V, ISO-DEP (ISO 14443-4), MIFARE Classic and Ultralight and all NFC Forum NDEF tags [46], Mifare SDK supporting Mifare Tags, ICODE and NTag [47], as well as the different library provided by the NFC reader manufacturers [48].

Although living labs were made to use NFC as a means of payment in 2008, it is only in the last two or three years that this technology has started to be put into use by different companies and banks. The main reason for this has been its use in security elements to facilitate payment credentials.

Not only do smartphones incorporate the security element of the NFC chip but they are also covered by their SIMs security element provided by different operators [49]. For this reason, software architecture was developed in 2012 designed to emulate the security element without the need for a physical chip within devices. This solution, called Host Card Emulation (HCE) [50], provides a secure channel of communication between the NFC reader, a mobile application installed in the device and the payment terminal. This has opened up the possibility for developing different NFC payment applications that have been implemented by MasterCard [51] and Visa [52] and the major banks of the world that use their own applications or properties such as Android Pay, Apple Pay, Samsung Pay, PayPal, etc.

Currently, NFC is being used in a large variety of applications: education [53,54], tourism [55], authentication [56,57], health [58], transport and ticketing [59,60], retail [61], marketing [62], etc. Its main development appears to be focused on the integration of NFC chips into all kinds of wearables [63] and even in the human body, to serve as a means of identification and as a trigger for other more complex services.

### 2.2. Bluetooth Low Energy (BLE)

Bluetooth Low Energy (BLE) devices, called BLE beacons, have generated great interest in developing solutions for the IoE setting, given that this enables the development of applications that allow for localization and tracking devices, which is to say people, both indoors and outdoors.

A beacon emits a signal from 50 to 70 m that can be detected by other compatible devices. Although this technology has existed since 1994, it wasn’t until June 2013 that Apple presented the iBeacon [64] specifications whereby Bluetooth technology became widely considered to be a facilitator of services based on localization.

The use of this type of BLE beacon offers more precision in indoor localization compared to other existing technologies within mobile devices e.g., GPS or WiFi. This characteristic allows them to be used in a wide variety of applications, geared towards the diffusion of information, the start of PoS (Points of Sale), user monitoring, etc. In many cases, it is necessary to link BLE technology with other technologies such as WiFi or NFC to be able to supply certain services of interest.

With the appearance of BLE technology, the opportunities for public and private organizations have increased in order to create new applications within the framework of smart cities. In this context, it is possible to proactively present relevant information for the consumer, increasing consumer engagement and interest with applications for beacon users, as long as the resulting notifications are not intrusive or overwhelming.

Most mobile devices that are currently made support BLE technology. With regards to the operating systems, the vast majority of devices with Apple iOS are equipped with software for the use of beacons. However, according to Google sources, 12.1% of Android devices are currently using the Bluetooth 4.3 version or above that offers total support for beacon use. In the case of Windows Mobiles, BLE support comes into use from version 8, which more than 50% of Windows mobiles use.

BLE technology emerged through the “Bluetooth Special Interest Group (SIG)” announcement of the Bluetooth Core Specification 4.0 [65], providing a continuous wireless connection (Bluetooth BR/EDR). This enabled applications such as audio streaming and connections using short bursts of long distance radio (Bluetooth LE). This made it an ideal solution for IoT because it reduces battery consumption of mobile devices as it does not need to be continually connected.

With this new BLE specification, it is possible to use chipsets in dual mode to support smart telephones that need to connect to other devices in BR/EDR mode (for example headphones) and in LE mode (for example, to connect to wearables or beacons). This architecture based on services uses the attributes protocol (ATT). All low level energy communication is made on a protocol of profiles, services and characteristics (GATT) [66].

This model allows the client and the server to interact in a structured way, transmitting data by means of regular packages of information with a pre-established format. Each type of beacon uses its own specification to give significance to the packages of data that it sends. Currently, three well-defined transmission protocols exist: (a) iBeacons; (b) AltBeacons and (c) URI Beacons (Eddystone open beacon).

#### 2.2.1. iBeacon Specification

This is a BLE protocol proposed by Apple [64] that has served as a reference for the rest of the existing protocols. iBeacon is an ownership standard that sustains a wide ecosystem of BLE products and software resources for developers who fundamentally target the Apple community. The iBeacon format is divided into five groups to structure the following information:

1. iBeacon Prefix (9 bytes): This represents a hexadecimal code that stores: (a) transmissions in LE mode, i.e., it only broadcasts-there is no connection; (b) specific manufacturing data; (c) Apple’s Bluetooth Sig ID and (d) a secondary identifier.
2. Proximity UUID (16 bytes): This is an identifier unique to the beacon, a standard 16 byte/128 bit (BLE UUID) that is often used within the company.
3. Major number (2 bytes): This identifies a sub group of beacons inside a larger group.
4. Minor number (2 bytes): This is used to identify each beacon individually.
5. TX power (1 byte): This is a value that indicates the intensity of the signal one metre away from the device. This parameter has to be calibrated for each device by the user or the manufacturer.

The normal mode of interaction through this protocol is to carry out a process of scanning to find the UUID, the most and least number of users with references to find information relating to the beacon in the database of the system that supports the application. The TX power field is used to determine what distance the mobile is from the beacon.

#### 2.2.2. AltBeacon

AltBeacons [67] is an open specification that emerges as a response to the iBeacon specification ownership. The AltBeacons specification offers the same functionality as the iBeacon with an open and configurable protocol. Of the 28 bytes that the specification uses for the notification message, 26 can be modified by the user.

In the AltBeacons specification, the first two unmodifiable bytes are the longitude of the notification data package and the manufacturing data. This protocol’s information packages are made up of:

1. AD LENGTH (1 byte): This is not modifiable by the user and indicates the package of data of the notifications.
2. AD TYPE (1 byte): This is also not modifiable and indicates the type of package.
3. MFG ID (2 bytes): This is the manufacturer’s Company code, assigned by Bluetooth SIG.
4. BEACON CODE (2 bytes): This is the AltBeacon notification code.
5. BEACON ID (20 bytes): This is the beacon’s unique identifier; the first 16 bytes have to be unique to the organization’s notifications. The rest of the bytes can be subdivided depending on the needs of each case of usage.
6. RSSI (1 byte): This is a value that indicates the intensity of the signal at one metre from the device. The number must be calibrated for each device by the user or the manufacturer.
7. MFG RESERVED (1 byte): This is reserved by the manufacturer to implement special functions.

#### 2.2.3. Eddystone Open Beacon

In 2014, Google launched the UriBeacon [68] project to investigate how BLE could be used to share URLs, resulting in the Eddystone [69] protocol that defines the format for BLE messages for beacon.

This format describes different types of frames that can be used individually or combined to use beacons that interact with various applications. The last version, Eddystone-EID (Ephemeral ID) [70], specifies a new type of frame that defines a secure cryptographic method so that information that is emitted by the beacons (including telemetry) can only be decrypted by authorized users.

The Eddystone protocol allows three types of packages to be emitted:

- The unique Eddystone-UID identification number of the beacon: This is similar to the UUID of the iBeacon. With this type of frame, push notifications or associated application actions can be unlinked.
- Eddystone-URL: This is the evolution of UriBeacon and enables URLs to be sent by the beacons. This information is collected by user devices and organized according to the proximity of the transmitting beacons.
- Eddystone-TLM (telemetry information): This is used to transmit data obtained from the sensors. This type of connection allows it to activate different actions, depending on different conditions, such as the temperature, air or noise pollution or humidity levels.

This protocol presents a great advantage over earlier ones, given that it not only transmits unique sequences (UID) that can be interpreted by certain applications but also Eddystone-URL package can send information that can be interpreted innately for any mobile device without having to install specific applications.

Table 2 shows a comparative study of the currently most popular and commercially used beacons on the market, including the web references on framework and software libraries for developing applications. As can be seen in the table, some of the characteristics of the products are not provided by the manufacturer and few devices are capable of supporting the three types of existing protocols, which means serious problems when choosing the right product.

Even if the signal range is similar in all of the devices, the battery life varies significantly, Estimote’s new devices being of the longest duration (7 years). The advantage of supporting iBeacon and Eddystone protocols is that they can be used with all mobile devices and navigators. The security of the communication is considered in almost all devices, even though it is an aspect that still presents problems [71]. There are, however, solutions based on secure signatures [72].

BLE is mainly used for indoor user/device localization for the construction of positioning and distribution maps based on fingerprint models. The use of BLE has been shown to be more efficient than the solutions based on WiFi technology because BLE is more susceptible to rapid fading and large fluctuations of RSS [73,74], obtaining precise measurements in indoor settings with the use of positioning algorithms, such as the combination of channel-separate polynomial regression model (PRM), channel-separate fingerprinting (FP), outlier detection and extended Kalman filtering (EKF) [75].

In these indoor settings, BLE has proved to be very useful in the monitoring or tracking of devices/users and the estimation of pedestrian dead reckoning (PDR). By working in isolation [76] or combined with WiFi and expanding algorithms based on fuzzy decision tree [77] and κ-Nearest Neighbours [78], the efficiency and low recognition and prediction error of routes is guaranteed. The PDR applications in BLE outdoor settings are normally used in combination with WiFi, GPS and even NFC [79].

The other main use of this technology is the use of the beacons as sources of information based on notifications by proximity [80], avoiding much of the need to install mobile applications in devices that are used to interact with the environment (Physical Web) [81,82,83] via smart services that supply the object with the same. In order to integrate physical elements in this context, models have been used that are based on externalizing tasks (crowdsourcing) that use beacons to identify and categorize information about the elements that are found following a similar approach to a scavenger hunt game [84].These combine with the BLE potential for the interior positioning allows personalized content to be provided to the user, depending on their interactions [85]. It is being applied within different sectors, such as tourism [86,87], banks [88], marketing [89,90], payments [91], traffic [92,93], etc.

### 2.3. Visible Light Communication (VLC)

Although wireless communication that uses light is not a new idea, over recent years it has caught the attention of researchers and companies as a possible solution to the expected demand from communication services. This demand for wireless communication is growing by 10% each year, meaning that by 2020 it will be the most sought-after by users. The existing broadband width (shared through WiFi, Bluetooth, cellular phone network, cordless phones) cannot meet the needs of user mobiles (using smartphones, tablets and new wearables) that require secure and fast data communication.

Given these expectations, attention has been turned back to electromagnetic mm long waves and frequencies above 100 GHz, which has been called Optical Wireless Communication (OWL). OWL presents certain advantages over radiofrequency (RF), such as the fact that equipment is cheaper, it does not affect health, it requires less energy and it uses a range that does not require a license (a range of 155–700 mm), etc.

Recently, pioneering ideas using light and data communication simultaneously emerged and it was proposed that the development of LED technology should only use a narrow bandwidth of OWL, comprising of between 380 and 780 nm and frequencies of between 4.3 Hz and 7.5 × 10^14^ Hz, meaning the spectrum of visible light that is detected by the human eye. This technology, called VLC (Visible Light Communication), has been developed in recent years by companies and institutions sponsored by the Visual Light Communication Consortium (VLCC) [94]. In 2001, the standard called 802.15.7-2011 (IEEE Standard for Local and Metropolitan Area Networks—Part 15.7: Short-Range Wireless Optical Communication Using Visible Light) [95] was published. In 2011, the term LiFi (Light Fidelity) appeared and the LiFi Consortium [96] was set up with the purpose of developing VLC as a means of high speed data communication.

**Figure 1:**
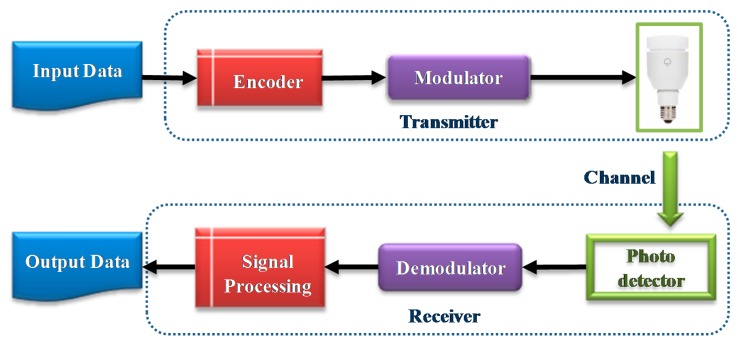
General structure of VLC components.

An architecture based on VLC infrastructure is formed by two elements: the transmitter and the receptor, as show in Figure 1, that communicate via a visible light channel [97].

The principal element of the transmitter is LED. There are various types of LEDs whose characteristics make them suitable for different applications [98]. From the point of view of emission, the most used LEDs are those that can emit white light (pc-LEDs that use a single chip) and generally in three colors, RGB, using three or more chips (Multi-chips LEDs). Other existing LEDs on the market are OLEDs (organic LEDs used for flat screens), μ-LEDs (micro-LEDs, developed specifically for VLC applications) and rc-LEDs (resonant cavity, aimed at improving coloured light emission to around 650 nm).

Although the multi-chip LEDs that emit in RGB have a wider bandwidth and offer the possibility of wavelength-division multiplexing (WDM) which provide a higher transmission speed than pc-LEDs, it is lower in cost than the latter, and new modulation techniques like multiple-input multiple output (MIMO), discrete multi-tone (DMT) and orthogonal frequency division multiplexing (OFDM), etc. enable speeds of 500 Mb/s [99] to be reached. Different algorithms are used to modulate the frequency of the LEDs for information transmission [100,101]: On-Off Keying (OOK), Pulse Position Modulation (PPM) and their different variants (EPPM, PAM, etc.), Colour Shift Key-in (CSK), Colour Intensity Modulation (CIM), Unipolar-OFDM (OFDM), etc. whose comparative characteristics have been studied [98].

The channel of communication that is used is a visible light spectrum, although there are different ways of establishing this channel between transmitter and receptor to create an optical link: with a direct Line of Sight (LOS) and without a direct line of vision and, depending on the degree of the directionality of the transmitter, receptor or both, can be directed, non-directed or hybrid [102].

The LOS channels are the most used systems for fast point to point communications. They allow for hundreds of Mbits/s or more, given that the signal does not suffer from distortion caused by multi-trajectories or noise from atmospheric light which is rejected to a large extent when a receptor is used with a narrow field of vision (FOV). The non-directed LOS configuration is more flexible when using transmitters with a wide beam as well as receptors with a wide field of vision. However, they need more emission potency to avoid disruption by the multi-trajectories of the signal. Hybrid LOS uses transmitters and receptors with a wide FOV, but not both.

The detectors can be different types: (a) Photodetectors (P-type, Intrinsic, N-type semiconductor layers), appropriate for settings in which the receptor receives a high intensity light from the transmitter; (b) Avalanche Photodiode (APD), appropriate for low level light intensity reception, although the latter has the disadvantage of creating more noise caused by a major photocurrent.

#### Advantages and Disadvantages

VLC offers many advantages for applications that require a wide bandwidth and communication security. Given that VLC is a secure communication technology, since it does not interfere with RF signals, it is therefore ideal for usage in sensitive indoor spaces, such as hospitals [103]. What is more, it uses green energy (LEDs) at low cost and with a conventional energy consumption of less than 20%, which, together with its ease of implementation in existing infrastructures in cities, allows for the potential development of smart cities [104].

However, VLC also has a series of disadvantages that are the subject of research and solution. These are:

- The transmission is influenced by blocking from objects, needing efficient recuperation protocols.
- The transmission of binary data is created by switching the LED on and off rapidly, losing the information over the width and phase of the wave.
- The LEDs flickering produces changes in the color of the light that can influence the mood of the users [105].
- Receiving pulsated light over long periods of time can cause users long term problems to mood and to the pupils of their eyes [106].
- It is still in the research and development stage. Companies have recently started commercializing their products but there is a lack of standardization or access to them.

Despite the disadvantages, the advantages of VLC for many applications are undeniable. For example:

- LiFi networks in indoor spaces (offices, hospitals, theatres, etc.), resolving saturation problems in areas where there is a high demand for WiFi [107,108], and emission problems of RF, which can interfere with sensitive environments, such as hospitals [109,110], chemical industries, etc. What is more, in indoor spaces, the LED infrastructure can be used as a system of localization, detection and guide [111], information, marketing, etc. [112,113,114].
- Traffic networks, supporting communication between signals and vehicles and vehicle-vehicle, allowing the exchange of information concerning status and conditions of the road, speed, routes and destinations, etc. [115,116,117].
- Underwater communication, given that it provides a higher speed and freedom from interferences than RF communication in this environment [118].

In recent years, very interesting and significant articles have been published about VLC and the possible applications of this technology. George et al. [101] review the latest advances in VLC and the most appropriate modulation techniques. Kumar and Lourenco [100] are researching the characteristics of LEDs for their application in VLC, proposing a basic architecture and some indoor and outdoor applications. Medina et al. [119] provided an extensive review of the characteristics, components, advantages and disadvantages and VLC technology applications.

Studies of VLC application in indoor spaces have invoked great interest due to its commercial interest and its relative ease of deployment. Thus, Schmid et al. [120] use LED light bulbs installed in a room that can communicate with each other and other VLC devices (e.g., toys, wearables, clothing). Wang et al. [121] propose an open source low cost (OpenVLC) to be used in smart indoor scenarios. Tian et al. [122] are developing a solution for indoor spaces with little or no light, achieving communication with LEDs that are invisible to the human eye.

Karunatilaka et al. [98] compare the use of RF and VLC, demonstrating that VLC is a more suitable proposal than RF by studying the parameters of filtering, equalization, compensation, and beamforming that should be adjusted to obtain stable communications without any loss thereof. Grobe et al. [123] are studying the potential uses of VLC in applications in indoor spaces.

The use of smartphones as transmitters and detectors of the VLC signal is one of the focuses of attention in order to enable this technology to have a wide deployment. The new CMOS detectors of these devices and their OLED screens are being widely tested. Kil-Sung et al. [124] use the flash and camera of Android Smartphones for the communication of data. The information (1s and 0s) is sent by means of OOK (ON/OFF Keying) codification, changing the flash of the camera to ON/OFF which is captured by the camera. Valisakis [125] and Hui-Yu [126] are studying the parameters that affect the use of Smartphones as receptors and propose various solutions (DynaLight y RollingLight, respectively) applicable to high and low range mobiles. Using an OOK codification with the analysis algorithm from Manchester and an improved algorithm of the image captured by the CMOs sensor in the camera to avoid problems with rolling shutter, synchronization, different speeds of sample devices, etc. distances of reception have been achieved between 20 and 120 cm, using LEDs multi-chips.

Although dozens of technology companies in Asia, Europe and USA, such as LVX System (Kennedy Space Center, Merritt Island, FL, USA), Nakagawa Laboratories (Tokyo, Japan), Oledcomm France LiFi (Vélizy Villacoublay, France), Taxan (San Jose, CA, USA), Panasonic (Barcelona, Spain), Casio Computer (Norderstedt, Germany), Fraunhofer (Munich, Germany), Philips (Amsterdam, The Netherlands), etc. have developed VLC devices and software, the hardware is still not available to companies that develop applications, meaning that the existing applications are living labs implemented by manufacturers of devices and LEDs.

Panasonic Corporation [127] has developed a VLC system called ID Light for indoor applications (museums, exhibition halls, hospitals, etc.) and outdoor (digital signage for tourism, transport etc.). Casio Computer Co., Ltd., has developed Picacamera so that consumers can receive information and has even incorporated this technology (Extrigger) so that motorists can receive direct messages whilst driving [128].

PureLifi (Edinburgh, UK) has developed LiFi-X, a system that allows the creation of networks of LEDs communication, reaching speeds of up to 40 Mbps in full duplex mode [129]. Acuity Brand (Atlanta, GA, USA) has developed a system ByteLight to control the location of clients in indoor spaces and for use in omnichannel marketing for larger surfaces [130]. LightBee uses VLC and Smartphones for solutions to identification and access control for people and vehicles [131].

One of the most complete solutions of VLC that exists on the market is one that was developed by Axrtek [132], called MOMO, that offers communication at 300 Mbs, using three color channels and software for the use of bidirectional transmissions of information in indoor and outdoor settings and in localisation and navigation.

Even though free software is available, in which VLC is used for different purposes (Arduino simple Visible Light Communication [133]), it is the result of prototypes from research. The availability of libraries or frameworks for the development of VLC applications is very limited. The manufacturers/distributors of products, such as PureLiFi [129], AccuityBrands [130], Qualcomm [134], Philips [135], etc. do not make these developments available for commercial use. However, Axrtek [132] own an API for the construction of applications of Smart Lighting and location-based services, and OpenVLC provide a platform for free software for the development of Lifi applications, which currently work with a small group of hardware components, thereby limiting its use in research [136].

## 3. Research Methodology

This section describes the research and development carried out with NFC, BLE and VLC technologies, the different projects and prototypes implanted in different cities around the world, solutions for entering, handling and analyzing data from connected devices and, finally, how the future of smart cities will be influenced by the development of these three technologies.

### 3.1. NFC, BLE and VLC in Smart Cities

Due to their different characteristics, maturity and development, the NFC, BLE and VLC technologies have had very different levels of success in the smart city projects emerging around the world.

As shown in Table 3, the different characteristics of speed, proximity, security, communication, cost, etc. means that the application of these technologies for smart city solutions are grouped into different areas.

To date there are several proposals about what the architecture should be for the areas and services in the smart city, for example, in [137] six categories of smart city services were proposed: people, governance, mobility, the environment and life; on the other hand in [138], a service structure based on a business model with eight categories was suggested. In [139] nine categories were identified: E-Government, E-democracy, E-Business, E-health and tele-care, E-Security, Environment, Intelligent Transportation, Communication, E-learning and E-education. In [140] the development of a smart city roadmap in Korea is described, and this approach analyzes the interdependencies between services, devices and technologies. Listed below are some of the applications of NFC, VLC and BLE technologies in the development of smart cities in these categories. This information was taken from the existing bibliography on current ongoing projects in some of the world’s cities, scientific papers about these technologies and their applications as well as the products developed by companies supplying these services.

#### 3.1.1. Administration, Governance

It is necessary for there to be a social service system that can intelligently manage the whole city, including such services as city planning for all-round, emergency and community management, as well as personalized attention to the individual citizen. With these services the government can compile and analyse data in real time, thus providing a rapid response and/or solutions for everybody [141].

Sahib [142] explores the different applications that NFC can provide for M-Governance, combining this technology with localisation technologies (GPS, BLE), and describes different applications for Government to Employees and Contractors.

BLE is a technology with a high potential for local government to be able to offer information and services to the public, including tourists and businesses, by means of a city guide to transport, maps, dangerous areas, pollution-free areas, recreation and public toilet areas, etc. Providing information on the location and actions of users enables immediate feedback, adding extra value to businesses and local administration [143].

Leading technological companies in NFC, BLE and payment methods have developed solutions to some local government administration management [144], such as tax payment, inventory and patient control, electronic voting, citizens’ suggestions collection, traffic control, etc. Until now there have been no solutions based on VLC.

#### 3.1.2. Education

The applications in this area have been centred on three main aspects: (a) identification and control of the presence of people; (b) identification and access to information in libraries; and (c) educational games [145,146,147,148,149,150,151].

NFC technology is the most used in these applications, associating tags with didactic spaces, students/teachers and books in libraries. Although BLE has been used in some prototypes [152,153,154], its cost and need for maintenance of devices has resulted in it being less viable than NFC.

To date, solutions based on VLC have not occurred, presumably due to the inexistence of LED infrastructures in learning centres, as well as the high cost compared to NFC. However, we consider that in the future, this technology will support these applications in education

#### 3.1.3. Healthcare and Social Services

Solutions based on BLE and NFC (and a combination of both) are being widely implemented. Medical equipment that enables smartphone and tablet pairing and the control and gathering of information [155,156], has allowed for identification of patients [157], vigilance and tracking of Alzheimer sufferers, dosage control of the blind and the elderly [158,159,160,161], remote assistance, etc. which are just some of the multiple solutions being implemented already.

State-of-the-art technologies are geared towards combining smart sensors with NFC chips to be implanted in patients [155]. In this way, analytical measurements can be taken in real time and at patients’ homes and be transmitted via smartphone to doctors.

Besides this, different solutions based on NFC have been proposed for charity organisations [162]. Users interact with readers present in PoS or Tags or wearables that are included in Smart posters or in clothing, and donations to charities can be made with a simple touch.

VLC would seem to be an ideal technology for the hospital environment due to there being no RF emissions, although, despite proposals having been published, there are no real implementations as yet. The greatest use of VLC in this area will be to substitute or share WiFi networks and those which communicate with medical instruments, given their transmission speed.

#### 3.1.4. Public Safety

Within this field we will consider security factors, both for people and assets. With regards to VLC, no solutions have yet been put forward in this field, despite its great potential. NFC has many implementations, e.g., in the Hollister Missouri School District and in many other learning centres [145] NFC is used for the control of access by school buses and to school entrances, as well as vehicle identification.

Intel has developed an ecosystem to manage security of devices, patients and sensitive information in hospitals via the use of NFC wristbands, and SoC NFC in medical instruments [163]. Different companies have developed new types of NFC Tags that guarantee consumer product authenticity [164] and food certification [165,166].

Some cities, such as Columbus (OH, US), have implemented citizen security systems based on BLE, such as security wristbands for public safety against perpetrators of domestic violence, etc. [167].

#### 3.1.5. Tourism, Recreation, and Culture

Tourism and culture, as well as events, purchasing and games are areas in which NFC and BLE have been implemented the most for a number of years. Some of the solutions that are present in many cities today, using BLE are: localisation and tracking of tourists or clients in indoor and outdoor spaces (supermarkets, museums, etc.) the use of NFC for identifying products [168], control of access to monuments and events and spreading information via the association of Tags to pictures in museums, tourist Smart Posters, statues, etc. [169]. BLE and NFC present themselves as ideal technologies for attracting clients/tourists and marketing campaigns, supporting systems that are sensitive in the context of aid to shipping, localisation, information spreading, coupon management, offers and tourist cards [170,171,172].

VLC promises great advantages in these areas, mainly indoors, as it enables the creation of applications in which control of locating and tracking of users is more precise and interactions are more natural. Thus, thanks to The Fraunhofer Heinrich Hertz Institute HHI, VLC has been used to create a LiFi network for events held on Mainau Island (Lake Constance) [173].

Given the high speed of VLC transmission compared to NFC and BLE and its capacity to locate users with little error, the main developments in this technology have been aimed at marketing solutions for attracting clients. These solutions are based on detecting the client in shopping centres, airports, etc. and passing on information to their Smartphones about offers, products, etc.

#### 3.1.6. Transportation, Mobility and Payment

Cities must take advantage of sensor networks and the IoT in order to establish smart traffic management systems, for which it is necessary to set up adaptable control and signage systems for the flow and volume of traffic.

Solutions have been put forward using the three technologies dealt with in this paper. For example, systems based on active RFID labels for automatic identification and storage information of vehicles in circulation [174], and in many cities around the world, public transport uses NFC for ticketing [175].

BLE is used to offer information services to passengers via Smart posters or beacons for public transport, train stations and airports, collecting information on travel, car parks, help for the blind [176], as well as vehicle-to-vehicle communication [177,178,179].

For VLC, the applications made for traffic control and management have aroused much interest, with a lot of research into vehicle circulation safety, vehicle-vehicle communication and signage, car park management, wireless communication networks for flights, thereby solving the problem of RF emissions produced by WiFi networks, etc. [180,181,182]. Until now these solutions have only been theoretically implemented in experiments [183,184,185].

NFC naturally prevails over other technologies in applications for payment and ticketing. Many world cities have incorporated contactless systems for payment on subways and buses, and the different NFC payment systems previously mentioned are a common method for users on all continents. Besides this, by using mobile devices, the latest developments are based on tokens and wearables, such as Jawbone, Apple Watch, Samsung Gear S2, Microsoft Band 2, etc., or even with NFC chips included in clothing [186].

Other means of payment are also available, based on BLE, such as PayPal, PowaTag, PassMarket, TruBeacon, LabWerk Beacons, etc. and ticketing-mainly in transport [187,188].

#### 3.1.7. Sustainability (Environment, Energy, Smart Buildings)

Smart Grid is one of the most important elements of the smart city. Management and energy management in cities and buildings will determine aspects of paramount importance to the sustainability of cities [189]. The Internet of things can be used for electrical power consumption monitoring, for example, in [190] a new approach based on a distributing agent technology and a novel quality of service (QoS) algorithm to transmit electrical information flows with multi-QoS constraints was proposed. Other proposals [191] were oriented to electromagnetic coupling between a broadband over power line (BPL) system and an HF communication system, using the moments method to determine the near-field pairing between the BPL and communication systems.

An evaluation of different radiofrequency protocols can be found in [192], which analyses the coexistence of different standards as KNX-RF Multi, Zigbee and EnOcean with BLE protocol available in mobile devices applied to Smart grids and the Smart home.

Although with few implementations so far, VLC will soon be the technology governments use for environmental conservation and reduction in energy consumption. Solutions will include those used in the cities of Fitchburg and Randolph (MA, USA) with street lighting and Smart Communications Systems [193]. The application of VLC enables underwater communication and provides better solutions to telemetry of buildings and land [194].

The application of VLC in Smart Homes and Smart Buildings is still in its initial stages, although some experimental proposals have been published, and there are as yet no standardised solutions. They are still costly and mainly deal with control of the electricity grid with the aim of improving the environment and saving energy, as in Philips Hue or Eldoled [195,196,197,198].

Beacons, NFC and sensors are used for many diverse proposals, such as the control and supply of water [199] or the Smart management of renewable energies and recycling depots [200]. As an example, the city of Barcelona has a green plan for reduction of CO_2_ emissions which includes the use of alternative energies, transport management and green construction policies [201].

In the development of Smart buildings, many NFC solutions have been put on the market which have demonstrated its natural usefulness in the control of accessing, blocking or activating locks, household appliances, etc. Due to its easy pairing with Bluetooth it facilitates communication with other electronic household devices [202]. This usefulness of NFC in Smart homes and Smart buildings is now being shared with the use of BLE, although the lack of a standard as mature as that of NFC means that these applications are of closed ownership, such as SmartThing, Beacon, BeaconAction, Insteon, etc., or OOrt which also uses LED technology [203].

### 3.2. Information and Knowledge in the Smart City

Several layers have been proposed for the smart city corresponding to different architecture proposals [138,140,201,204,205,206]. In order to create a more appreciable and measurable environment, with greater interconnection, interoperability and increasingly smarter, a minimum of three layers of architecture must be considered. These three layers are the following: (a) a layer of perception and applications; (b) a network layer and (c) the application/intelligent layer.

In this type of architecture the data layer is considered as part of the application/intelligent layer [204,206]. However very few of the solutions described in the previous section for smart cities using NFC, BLE and VLC technologies contain the three layers necessary for an organised smart city.

The perception layer identifies real world objects by extracting environmental information via the use of sensors and receptors, such as Tags and NFC readers, BLE beacons, or VLC systems. The network layer guarantees the transmission of information obtained in the perception layer using mobile networks (3G/4G, WiFi, Mesh, WiMax, LiFi, etc.) and Smart Processing Centres. In order to do so, it is necessary to develop and integrate these layers to be able to analyze and process all the data and information coming in, through cloud computing, applying matching-learning techniques to infer the necessary knowledge for smart city development.

In a smart city environment where everything is connected with an IoT architecture, such a large volume of data is generated that it has become one of the major challenges for Big Data, and there are several studies that show that the volume of data generated in recent years exceeds a zettabyte [207,208,209,210].

The EU estimates that for this year there will be around 1.7 million billion data bytes per minute [211], and by the year 2020 it will have reached 35 zettabytes [212], distinguishing between data “about things” and data “generated by things”, the former defining state, location, identity, etc. the latter referring to data generated or received by things [213].

In order to analyse and process large volumes of data over several years, various matching-learning techniques have been proposed, such as clustering [214], random sampling [215], data condensation [216], divide and conquer [217], and incremental learning [218].

Given the complexity of “information generated by things”, in order to apply these techniques, it is important to reduce the complexity of incoming data, for which features selection [219,220] or instance selection [221] are used.

Data mining and knowledge extraction in the IoT are carried out with the following steps: selection, pre-processing, transformation, data mining and interpretation and/or evaluation [222]. Of these steps, data mining is the most important due to the fact that it is the process which enables the extraction of patterns to model and infer knowledge.

The data mining techniques most used in the IoT are clustering, whose aim is to classify patterns with supervision, without supervision (the patterns are unlabelled), Association Rules whose aim is to find events of pattern entries with no order of occurrence, and Sequential Patterns, whose aim is to find a determined occurrence in patterns.

Regression models constitute another technique widely used for Big Data analysis in smart cities [223]. This technique builds a continuous function between a dependent variable and one or several independent variables. The dependent variable is selected according to the problem in question, and it is also necessary to use an error margin to adjust the model, which is usually the difference between the value predicted from the regression and the real value.

Within the ecosystem of a smart city and the IoT the regression models have been applied to the prediction of the necessary loads on the smart grids, enabling the acquisition of a global prediction via the sum of predictions over the composition of individual loads [222]. They have also been used to predict energy consumption in smart cities [224] to be able to predict water demand from different information sources [225,226,227], to evaluate indexes in the monitoring of smart cities [228], journey-planning taking into account traffic incidents [229] and in the prediction of parking space availability [230].

Clustering methods have been used to improve communication network behavior by using information coming from wireless sensors [231,232,233], which can predict behavior of inhabitants using data from networks and Smart home sensors [234], for housekeeping using vacuum sensors [235], in farm management with GPS and field sensors [236,237] and to analyse the behavior of members of a social network using information from RFID sensors, smartphones, BLE devices, etc. [238,239].

Classification methods have been widely used in smart cities for predicting possible actions of inhabitants from data stored in video cameras, microphones, RFID sensors, wearable kinematic sensors, [240,241,242,243,244,245,246], etc. These data mining techniques have also been used for traffic control in cities using information from GPS, mobile devices and sensors placed in vehicles [247,248] and for recognition and identification of devices using RFID sensors [249].

The analysis of pattern frequency is of special interest for interpreting all stored information obtained from interactions occurring in smart cities. This data mining technique has been used, for example, in the management of RFID tags located in and around the city [250], and for predicting the behavior of inhabitants by analysing information received by RFID sensors [251,252,253,254].

Finding efficient solutions to the Big Data problems arising in smart cities with everything connected in an IoT model will enable governments and society itself to transform, as well as finding and explaining relations among inhabitants, institutions and things, which is information that is currently hidden within an immense volume of data that is constantly growing.

These Big Data solutions will improve public services, for example in the current data crossing of incidences with humidity, traffic and temperature sensors, which enables a more efficient use of irrigation systems, waste management, transport, etc. With regard to public safety, there are global maps drawn from sensors located conveniently around the city showing security guards all the alerts and alarms, such as earthquakes, storms and general contingencies.

The security system in New York has several transparent and connected information sources for operators, including CCTV cameras, traffic lights, industrial systems, humidity sensors, presence sensors, trespass-detecting systems, access security systems mobiles, computers, etc.

The transfer of data from credit card transactions with information about the holding of events enables the financial contribution of such events in the city to be known, as well as the relations between participants and local businesses [255].

A daily increase is seen in the use of gadgets or wearables for the purpose of measuring different biometric factors of people, such as weight, physical activity, calorie consumption, commuting habits and sleeping habits, etc. All this stored information, if used correctly as part of Big Data solutions, will enable the transformation of the current model of personalised medicine into one of preventive medicine. According to primary studies in the US it is hoped that healthcare costs can be cut, as well as errors in diagnosis and in the prescribing and administering of medicines [256].

Tourism is another area that benefits from Big Data solutions in smart cities. This new focus enables data analysis of tourists in real actions rather than just from polls and surveys. Starting with obtaining objective information from the place of departure, length of stay, traveling and tourist movements, it is possible to improve decision-making processes, increase the efficiency of negotiation processes, increase client attraction and the focus of trade.

### 3.3. Future Trends of NFC, BLE and VLC in the Smart Cities

**Figure 2:**
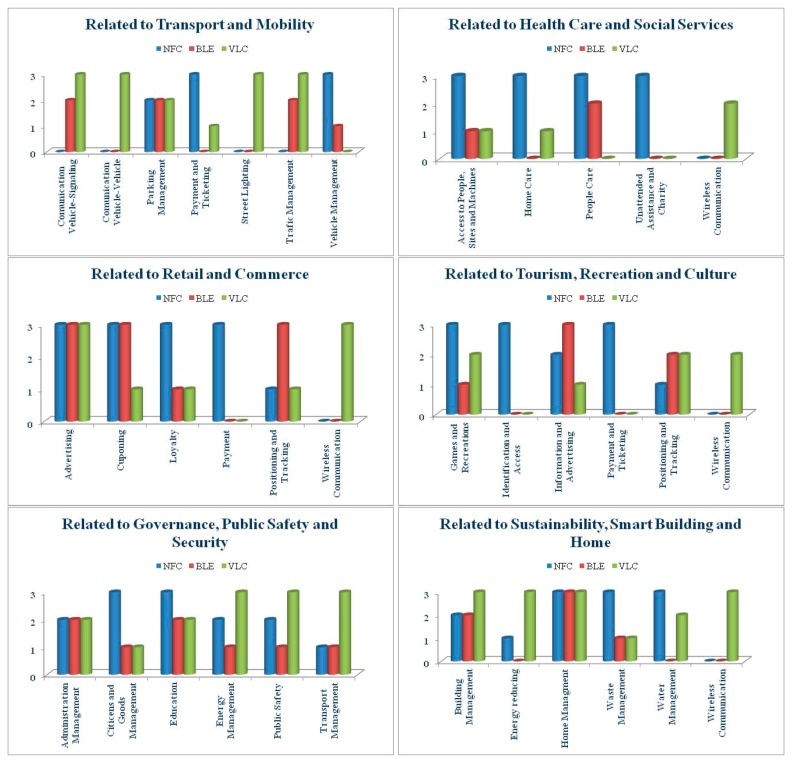
State of art and visionary evolution of NFC, BLE and VLC technologies.

In this section we describe our vision of the participation of these technologies in the near future, based on their current status, government and business proposals on offer and the status of existing applications [257,258,259] (see Figure 2).

Batty et al. [260] give a proposal for future smart cities in each area of their development, network integration and information in large databases that are shared and accessed by the public. The combined use of communication technologies, such as those dealt with in this paper, are the basis to their proposal.

Anttiroiko [261] proposes the combination of RFID technologies, ubiquitous sensor network (USN), GPS, WiFi, etc. in order to create a fairer and friendlier city, giving as an example the initiatives in Korea. Cheng et al. [79] propose the combination of NFC and BLE for systems of locating, tracking and information and they apply their proposals to indoor and outdoor settings.

Wirtz et al. [262] show the advantages of combining NFC, BLE and VLC in the IoT, making it possible to discover units and things in the setting and their integration in a common framework.

There are many reasons why NFC will be part of smart cities: (a) it is easy and intuitive to connect with devices and machines; (b) it is not intrusive given that the connection is always made by the user; (c) it is safe because of the distance of connection and the existing standards and specifications; (d) it is flexible given that it enables connection with and control of any object, accessing information and services with a single movement; (e) it is low in cost and energy consumption.

NFC will maintain its prevalence in ticketing and payments, and in its support of all types of sensors and instruments used for the measurement of patient parameters. It will be the link between “things” and the “cloud”, via the new IC chips, the NFC-X standards, the registering and maintenance of objects (IoT) and devices (IoD) that make up the settings and their pairing via BLE, VLC and other communication technologies [263,264].

Although the transition of cities to LED technology is slow due to the cost, the commitment to sustainability in cities and the clear capitalisation of this transition will ensure a rapid change. It will be accomplished by creating Light Sensory Networks (LSN), using LED infrastructure, which will obtain information in real time about the physical environment [265]. Analysis of these large databases will allow, via the LSN, transmission of information to the general public on the ”state of the world”, creating new types of services and applications in the smart city.

These applications, as with those mentioned above, for transport and traffic, safety, buildings, mobility, flights and underwater communication, health and hygiene, games, etc. will combine several technologies, given that it is still necessary to resolve the challenges of existing integration [266,267].

Figure 2 represents our vision of the relative implication of NFC, BLE and VLC technologies over the next few years in the different areas of development of smart cities. It is a relative vision taking into account the current situation and existing projects for each of the three technologies.

#### 3.3.1. Related to Transport and Mobility

Street lighting is one of the great challenges facing city sustainability, due to pollution generated and the cost to governments, for which the use of LEDs and sensors for street lighting will also provide a sustainable solution by offering Smart services to the public and potential solutions to retailers to access their clients via LiFi-WiFi networks [268].

Likewise, management of traffic and car parks will be another priority to resolve. A Smart Lighting infrastructure, based on VLC, road signage with LEDs, the use of BLE beacons and cameras with software using new and powerful algorithms for vision and image treatments for management and control of traffic will all be supported by VLC vehicle-vehicle communication, providing data in real time on traffic status and helping to keep driving safe [268].

NFC is already a reality in vehicles, allowing access and keyless ignition with a mobile device and different types of wearables. BLE will be used safety and locating systems.

Payment via NFC for public transport and parking is one of the most piloted actions under development and will soon be a generally accepted reality. The use of wearables and tokens to replace credit cards and even mobile phones will soon be widespread.

Although payment systems based on BLE have been started, they are experiencing little or no implementation. VLC as a means of payment for public transport at toll booths has been researched, but, despite its multiple advantages, is still in its early stages.

#### 3.3.2. Related to Health Care and Social Services

An aging population and the cost of healthcare call for a change towards home visits, and NFC technology plays and will continue to play an important role in this. Skin tags and mobile instruments for measuring clinical parameters and medical conditions, e.g., sugar levels, cholesterol, blood pressure, chronic obstructive pulmonary disorder (COPD), systems to help and control administration of medicine etc. will play a fundamental role in health care outside hospitals.

NFC will also be the predominant technology in patient identification and personal healthcare in home visits, as well as in hospitals for control of access, instruments and patient records [269].

BLE technology has shown its efficiency in locating patients in hospitals and homes for the elderly, as well as in helping the elderly and disabled, in the same way as NFC technology.

The development of VLC in this area will depend on the capacity of governments to take on the relevant costs thereof. Its evolution will determine the implementation of LiFi networks in hospitals and the development of sensors in medical instruments to enable communication between doctors and patients via mobile devices.

#### 3.3.3. Related to Retail and Commerce

There is a clear tendency towards businesses and governments using NFC for offers and coupons, although BLE has shown its advantages for reaching a wider range of smartphone users, as seen in the strategic changes of companies such as Proxama or RetailMeNot [270].

Locating people and following them in indoor spaces, important for retailers, will be one of the challenges in which several technologies will coexist, based on the cost of their infrastructure, accuracy required, user need for information, services on offer, etc. The solutions that are based on the combined use of BLE and VLC for locating and tracking, and those based on NFC as producer of services, are already in use in large department stores and museums, etc. [271].

While it is hoped that in 2016 the number of users of NFC for payments reaches 148 million, in 2020 it will multiply by 5 [272], and the obsolete infrastructure of the use of SIM operators and security elements of devices will be substituted by HCE technology and wearables.

It is estimated that around 23 billion transport tickets will be bought via smartphones in 2020. Here, NFC and BLE will be the technologies used, although the maturity of NFC, tokens and added security that it brings will make it more popular [261].

#### 3.3.4. Related to Tourism, Recreation and Culture

The tourism and culture sector is where NFC y BLE [170,171,260] have undergone and will undergo the biggest development. Solutions include tourist cards, ticketing and identification for events, transport, monuments, etc. guide systems, location in museums, routes, etc. advertising systems, maps, local information, etc.

For its part, the presence of VLC in tourism and culture will continue advancing parallel to the development of LED infrastructure in cities, although for indoor setting solutions, such as in museums, where it already exists, it will be the predominant technology.

Displays, TVs, etc. and many games incorporate LEDs resulting in VLC being present in many interactive games for children and adults, enabling the creation of interactive communication environments [273,274]. Even though BLE has not had any presence in this area, NFC, due to its simplicity, will increase its presence therein, enabling communication between the real world and the virtual one of games [275].

#### 3.3.5. Related to Governance, Public Safety and Security

Within these aspects of the smart city, all other areas are implicated, mainly, the development of Big Data and the providing of information thereof to the general public, as is happening already in cities around the world [276].

In transport, as has been mentioned, NFC will be mostly for tickets and payments and, together with BLE, for citizen support, while VLC is the most appropriate technology for the management of transport infrastructures. The improvement in sustainability of cities via the implementation of LEDs for energy saving and pollution reduction will enable these solutions to be developed, and will also serve to improve capacities for wireless communication of data and control measures of security in cities via Light Sensor Networks (LSN) [264].

A Light Sensor Network (LSN) based on VLC can provide both transport capabilities to streaming video and audio and real time information to be sent to the cloud database to be analyzed, generating, if necessary, corresponding alerts. Via LSN of VLC much local government management can be improved: (a) in transport (control of vehicle and people density, identification of vehicles and licenses, etc.); (b) in the security of buildings and urban areas (detection of movement, perimeter protection, break-ins, etc.); (c) in peoples’ safety (aggressive behavior, detection of alarms for burglary, shootings, fires or explosions, etc.); (d) in education centres, hospitals, government office buildings, etc. (identification of vehicles, movement and access of people and vehicles, etc.). Although these actions require the implementation of LED and LSN technology, their deployment does not require costly engineering work, special permits or complex logistics.

For its part, NFC plays, and will continue to do so, an important role in safety management, identification of people and products, forgery and fraud. It does so with the use of secure chips and especially the supports and materials connected to sensors associated with products and even implanted in people. The use of NFC and BLE, which is more and more widespread as a means of public participation for waste/garbage collection, information desks and alarm alerts, etc. enables the general public to inform authorities of any incidents and those authorities to provide information in return, as well as improving the management of public services.

#### 3.3.6. Related to Sustainability and Smart Building and Homes

Building management such as smart meters and monitoring devices can help monitor and manage water consumption, heating, air-conditioning, lighting and physical security. This can allow the development of Smart utility grids with bidirectional flow in a distributed generation scheme requiring real-time exchange of information [10].

It is estimated that cities are responsible for between 60% and 80% of the world’s energy use. Optimising delivery and consumption is vital. Smart grid technology aims to tailor the generation and supply of energy to user consumption, thus increasing efficiency, reducing costs and environmental impact.

In particular, consumer “Smart Meters” and sensors, equipped with IP addresses, can communicate information about energy usage patterns to the supplier, as well as allowing end-user control. This can help manage real-time demand, and even provide advice to consumers about usage habits.

Buildings, both residential and commercial, provide an important opportunity to optimize energy consumption and enhance the wellbeing of residents and workers. Smart buildings, particularly office environments, are able to leverage Smart Grid technologies to influence energy supply and consumption by controlling lighting, climate control and IT. They can even provide electric plug-in stations for employees to recharge their cars while at work [277].

NFC will be present in many actions for the sustainability of cities, enlarging the existing projects in Berlin or Groningen in which NFC Tags and sensors are used for improved waste collection and recycling [278].

Furthermore, BLE technology holds a promising future in the smart management of renewable energies and recycling depots [200]. VLC will be widely used in the construction of building energy management systems in buildings where lighting is a major component of electrical energy consumption [279,280].

## 4. Conclusions

This review article shows the importance of technologies (NFC, BLE, VLC) for attaining the integration of all objects and sensors that make up the Internet of Things in the main areas of a smart city. To achieve this goal, it is necessary to integrate these three technologies with others for Machine to Machine (M2M) communication such as LoRaWAN, Sigfox, Weightless, LTE, and 5G, considered to be one of the key enablers for the provision of advanced applications and services in smart cities.

In addition, the new technologies described here are vital to integrate all the elements that surround us. Connected things by M2M protocols, such as automated teller machines and airline check-in machines, have previously existed. However, new devices, and especially ordinary passive objects around us are being augmented and integrated into the IoT through emerging technologies like NFC, BLE and VLC. A great social transformation is foreseen in which information and communication technologies will create new businesses and opportunities via more sustainable use and management of resources, whilst also guaranteeing city planning in a visual, measurable and intelligent way.

In a smart city environment NFC is a low cost and energy saving technology useful to connect real objects with machines, it is not intrusive given that the connection is always made by the user. Its flexibility allows the control any object, accessing information and services with a single click.

The advent of BLE technology increases opportunities for public and private organizations to create new indoor/outdoor localisation applications within the smart cities. In this context it is possible to present relevant information proactively to consumers, increasing the commitment and interest of consumer applications by using beacons, provided that the resulting notifications are nonintrusive.

VLC will replace some of the current uses of NFC and BLE. Its development will depend on the standardization of LED products and chips as well as the availability of software for public use which will enable the development of libraries and frameworks and allow the generalised construction of applications for this technology.

There are many challenges that the future development of these three technologies must address. In the case of NFC technology, the first challenge that must be addressed is: getting known. Today there are many users with this technology on their mobile phones who do not know how to use it. The maximum data transfer rate for NFC is 424 Kbits/s making it inappropriate for large quantity of data transfers. Although this technology is safe, it must be improved to prevent, for example, attacks relayed by employing two special communication devices between the victim reader and the victim tag. These security problems represent a major challenge in BLE and VLC technologies where communication is performed at a greater distance.

Another challenge for the attention of BLE technology is the limited range problem; the range is directly dependent on Broadcasting Signal Power. An increase in signal power makes BLE devices less energy-efficient. Moreover it is necessary to improve accuracy in determining proximity to a BLE device.

VLC is a newer technology and thus more challenges there are to address, such as providing an upstream communication channel, the development of LEDs with special features for VLC, search for efficient ways to communicate when the lights are off, finding more efficient mechanisms to avoid interference from sunlight, the proposal of new and more efficient modulation schemes, etc.

The application of data mining techniques to store information from sensors and mobile devices and augmented objects in an environment of IoT helps to develop more user-friendly systems that contribute to a smart city. In this area one of the most important challenges to be solved is the treatment and analysis of large volumes of information from very heterogeneous sources.

## Figures and Tables

**Table 1:** Comparison between the most used tag types.

Characteristics	NFC Tag Types								
	NTAG203	NTAG210/2	NTAG213/5/6	Mifare 1k/4k	Ultralight/C	Topaz	Desfire EV1	ICODE Series	NTAG I^2^C
Standard (ISO/IEC)	14443A	14443A	14443A	14443A	14443A	18092 21481 14443A	14443A 7816-4	14443B-3 15693 18000-3	14443A
Tag Type	Type 2	Type 2	Type 2	Classic	Type 2	Type 1	Type 4	Type 5	Type 2
Memory	168 bytes	80/164 bytes	180/540/924 bytes	1/4 Kb	64/192 bytes	120 bytes	2/4/8 KBytes	256/896/1280 bits	888/1094 bits
Available Memory	137 bytes	48/128 bytes	137/496/879 bytes	716 bytes	46/137 bytes	96 bytes	2304/4864/7936 bytes	256/896/1280 bits	896 bytes
Encriptation	No	No	No	Yes	No/Yes	No	Yes	Yes	No
Password protection	No	No	Yes	No	No	No	Yes	Yes	No
Hard protection	Yes	Yes	Yes	Yes	Yes	Yes	Yes	Yes	Yes
Scan counting	No	No	Yes	No	No	No	No	No	No
UID ASCII mirroring	No	Yes	Yes	No	No	No	No	No	No
Transmission speed	High	High	Very high	Slow	Medium	Medium	Medium	High	High

**Table 2:** Comparison of the most popular commercial beacon.

Characteristics	StickNFind	Estimote	Kontakt.io	RedBearLabs	Radius Networks	KST	Proximity	Blueup	Glimworm	Qualcom	GeLo
Devices types	Beacon	Beacon and Stickers	Beacon	Beacon B1	Beacon and USB Dongle	Particle	Beacon X/O	Beacon mini, Beacon maxi, USB	Beacon	Gimbal Beacon	Beacon
Protocol	S-Beacon	iBeacon and Eddystone	iBeacon and Eddystone	iBeacon	iBeacon , AltBeacon, Eddystone	iBeacon and Eddystone	iBeacon	iBeacon and Eddystone	iBeacon	Gimbal	GeLo
Power type	Battery	Battery	Battery	Battery	Battery/external	Battery	Battery	Unknown	Battery	Battery	Battery
Operating life	1–3 years	7 years	Unknown	1 year	Unknown	6 months	5 years	Unknown	1 year	Unknown	2 years
Radio range	50 m	70 m	Unknown	50 m	Unknown	50 m	60 m	Unknown	2 m, 20 m, 50 m	50 m	10 m
Certifications	FCC, CE, AS4268	Pending	Unknown	Non reported	Unknown	FCC, IC	Unknown	Unknown	Unknown	Non reported	Non reported
Chipset	nRF51822	nRF51822	Unknown	CC2540	Unknown	nRF51822	Unknown	Unknown	CC2450	Unknown	Unknown
Cloud management platform	Yes	No	Yes	No	Unknown	No	Unknown	Unknown	Unknown	Yes	Yes
Firmware updatable	Yes	Yes	Unknown	Yes	Unknown	Yes	Unknown	Unknown	Unknown	Yes	Unknown
Firmware update secured	Yes	Yes	Yes	No	Unknown	Unknown	Unknown	Unknown	Unknown	Yes	Unknown
Support encrypted password security	Yes	Yes	Yes	No	Unknown	No	Yes	Yes	Unknown	Yes	Unknown
Configurable radio output power	Yes	Yes	Unknown	Yes	Unknown	No	Unknown	Yes	Unknown	No	Unknown
Configurable measured power (RSSI)	Yes	Yes	Unknown	Yes	Unknown	No	Unknown	Yes	Unknown	Yes	Unknown
Default Beacon broadcast rate	1000 ms	200 ms	Unknown	250 ms	Unknown	Unknown	100 ms	100 ms	100/1285ms	Unknown	Unknown
Configurable advertising interval	Yes	Yes	Unknown	Yes	Unknown	No	Unknown	Yes	Yes	Unknown	No
Configurable UUID	Yes	Yes	Unknown	Yes	Unknown	Yes	Unknown	Yes	Yes	Unknown	No
Configurable MajorID, MinorID	Yes	Yes	Unknown	Yes	Unknown	Yes	Unknown	Yes	Yes	Unknown	No
Sample app	Yes	Yes	Unknown	Yes	Yes	Yes	Unknown	Unknown	Unknown	No	Yes
iOS SDK	Yes	Yes	Yes	No	Yes	No	Yes	Yes		Yes	Yes
Android SDK	Yes	Yes	Yes	No	Yes	No	Yes	Yes	Unknown	Yes	No
URL (information, frameworks and libraries)	https://www.sticknfind.com/Beacons&iBeacons/	http://estimote.com/	https://kontakt.io	http://redbearlab.com/iBeacon/	http://www.radiusnetworks.com/	https://kstechnologies.com/particle/	http://roximity.com/platform/	http://www.blueupBeacons.com/	http://glimwormBeacons.com	http://www.gimbal.com/	http://www.getgelo.com/Beacons/

**Table 3:** Comparison of the characteristics of NFC, BLE and VLC technologies.

Characteristics	NFC	BLE	VLC
Standard	ISO/IEC 14443 A&B, JIS X-6319-4	Bluetooth Core Specification 4.0/4.1/4.2	IEEE 802.15.7, JEITA CP-1223
Band width	13.56 MHz	2.4 GHz	400 nm (750 THz)–700 nm (428 THz)
Frequency regulation	Regulated. Limited band with	Regulated. Limited bandwidth	Unregulated
Transmission rate	424 Kbits/s	300 Kbits/s	Mbs-Gbs
Transmision distance	<10 cm	<70 m	<120 m
Transmision security	High	Low	What you see is what you send
Technology maturity	Mature	Mature	Little mature
EM interferences	Yes	Yes	No
Infrastructure	Access point	Access point	Lighting
Vision line	Yes (Contact)	No	Yes/No
Communication	One to one	One to many	One to many
Energy consumption	Very low	Medium	Low
Energy efficiency	None (Tags). Low others	Battery (average 2 years)	LEDs (low)
Green sustainability	High	Low	High
Coverage	4–10 cm	50–70 m	20–50 m
Interaction mobility	None	Wide	Limited
M2M transmission	Bidirectional	Bidirectional	Bidirectional
Infra2M Transmission	Bidirectional/Unidirectional	Unidirectional	Bidirectional
Dangerousness	None	Yes (RF emision)	Eyes /Frame of mind
Environment conditions	No effect	Reduction of the range	Interferences
Noise sensitive	No	Other users	Environment conditions
Operating system	Android, Windows, Blackberry	iOS, Android, Windows, Blackberry	In development
Localization	Consumer control	Consumer respond	Both
Privacy	No intrusive	Intrusive	Intrusive
Security	Secure	Unsucure/Secure	Unsecure
Cost	<$0.10	<$20	n/a
